# Does the recovery of serum FSH influence the ovarian sensitivity index in the follicular-phase depot GnRH agonist protocol?

**DOI:** 10.1186/s12884-026-09246-3

**Published:** 2026-05-19

**Authors:** Xiaoning Wang, Shanshan Zhu, Kui Fu, Yifan Han, Xin Chen, Changjun Zhang, Ying Zhang

**Affiliations:** 1 Reproductive Medicine Center, Renmin Hospital, Hubei University of Medicine, 39 Chaoyang Zhong Lu, Shiyan, Hubei Province People’s Republic of China; 2 Hubei Clinical Research Center for Reproductive Medicine, Shiyan, People’s Republic of China; 3 Biomedical Engineering College, Hubei University of Medicine, Shiyan, People’s Republic of China; 4 Biomedical Research Institute, Hubei University of Medicine, Shiyan, People’s Republic of China

**Keywords:** FSH, In vitro fertilization, GnRHa, Ovarian sensitivity index

## Abstract

**Background:**

The follicular-phase long GnRHa protocol is a common protocol in COH. This study mainly analyzed the recovery of FSH levels on the day of Gn initiation compared with basal FSH levels on the day of downregulation and studied the correlation with the OSI to discover a new monitoring target for COH.

**Methods:**

A total of 2286 patients who underwent IVF/ICSI with the follicular-phase depot GnRH agonist protocol from January 2018 to January 2023 were stratified into cohorts. The primary outcome was the OSI in patients who received the follicular-phase depot GnRH agonist protocol. The GnFSH/bFSH ratio was used to represent FSH recovery. The enrolled patients were divided into 3 groups according to GnFSH/bFSH tertiles, and cycle characteristics and outcomes were compared. The relationship between the GnFSH/bFSH ratio and OSI was analyzed using smoothed curve fitting to determine the threshold or saturation point for the study population. Then, the relationship between the GnFSH/bFSH and OSI was tested with a multivariable linear regression model after adjusting for potential confounders.

**Result(s):**

After adjusting for potential confounders, when the GnFSH/bFSH ratio was less than 0.8, the OSI increased with increasing GnFSH/bFSH values (β = 2.96, 95% CI = 2.37, 3.56, *P* < 0.0001). However, there was no significant relationship between GnFSH/bFSH and OSI values when the ratio was higher than 0.8 (β = 0.19, 95% CI= -2.02, 2.39, *P* = 0.8686). In the multiple linear regression model, after full adjustment, each one-unit increase in the GnFSH/bFSH value was related to increased OSI values (β = 2.59, 95% CI 2.10, 3.09, *P* < 0.0001). In GnFSH/bFSH tertile groups, patients in T2 (0.37–0.56) and T3 (> 0.56) had increased OSI values (β = 0.49, 95% CI 0.24, 0.74, *P* = 0.0001 and β = 1.25, 95% CI 0.99, 1.51, *P* < 0.0001, respectively) when compared with patients with values in T1 (< 0.37).

**Conclusion(s):**

The recovery of serum FSH (GnFSH/bFSH) after pituitary suppression is an independent risk factor for increased OSI values in women undergoing the follicular-phase depot GnRH agonist protocol.

**Supplementary Information:**

The online version contains supplementary material available at 10.1186/s12884-026-09246-3.

## Background

In the last 40 years, assisted reproductive technology (ART) has developed rapidly and has become an important method for the treatment of infertility. Sunderam showed that despite a gradual increase in clinical pregnancy rates with ART, the live birth rate was only 38.1% for each in vitro fertilization-embryo transfer (IVF-ET) cycle [1]. Controlled ovarian hyperstimulation (COH) is one of the most important techniques in ART [2]. Due to individual differences, individuals have different ovarian responses to COH, and two adverse outcomes (high or low ovarian response) may occur after COH [3]. Therefore, it is very important to accurately predict the ovarian response after IVF/ICSI (in vitro fertilization/intracytoplasmic sperm injection) [4].

In current studies, age, AMH (anti-Mullerian hormone) levels, InH B (Inhibins B), AFC (antral follicle count), basal FSH (follicle-stimulating hormone) levels and other indicators are commonly used to predict ovarian responsiveness [5]. Some scientists have proposed using the ovarian sensitivity index (OSI) to predict ovarian response [6], which is defined as the number of oocytes retrieved/total amount of gonadotropin. Subsequently, the OSI was confirmed to be related to the AMH level and AFC [7, 8]. Pan [9] showed that when the OSI value was lower, the ovarian sensitivity and pregnancy rate was lower.

GnRHa is a commonly used drug to prevent early LH surge during IVF/ICSI [10]. In China, the standard GnRHa long protocol has been widely used due to its high fresh embryo transfer (ET) clinical pregnancy rate and stable clinical pregnancy rate [11]. The mainstream protocol in our center is also the follicular-phase depot GnRH agonist protocol. It is undeniable that the protocol has many advantages include higher clinical pregnancy rate [12] and higher ongoing pregnancy rate [13]. Besides the follicular-phase depot GnRH agonist protocol may improve endometrial receptivity [14], and be more friendly to patients with repeated IVF failure [15]. Gonadotropin (Gn) is an essential drug in the process of ovulation induction. The dosage of Gn is related to the patient’s age, body mass index (BMI), ovarian reserve function, hormone levels, personal and family history, and lifestyle and living environment. To ensure the efficacy and safety of Gn in COH, we hope to find convenient and sensitive indicators that can help to predict the OSI. Therefore, this study mainly focused on the population undergoing the follicular-phase depot GnRH agonist protocol by analyzing the recovery of FSH on the day of Gn initiation compared with the basal FSH level on the day of downregulation and studying the correlation with the OSI to discover a new monitoring target for COH and to better guide reproductive doctors to predict the effect of COH and select the appropriate Gn dosage.

## Materials and methods

### Study inclusion and exclusion criteria

From January 2018 to January 2023, patients who underwent IVF/ICSI in the Reproductive Medicine Center of Shiyan People’s Hospital Affiliated with Hubei University of Medicine were analyzed. All patients were treated with the follicular-phase depot GnRH agonist protocol. A total of 2286 patients were included in this study according to the inclusion and exclusion criteria.

The inclusion criteria were as follows: all patients who met the diagnostic criteria for infertility, had undergone their first IVF cycle, were treated with the follicular-phase GnRH agonist protocol, were aged < 35 years, and had an FSH level < 25 mIU/ml.

The exclusion criteria were as follows: patients with severe medical diseases such as hypertension, diabetes, gastrointestinal diseases, thyroid disease, hyperprolactinemia, and congenital adrenal hyperplasia and those who underwent PGT.

### Ovarian stimulation in IVF

In the follicular-phase GnRH agonist protocol [17], the patients received a single 3.75-mg intramuscular injection of long-acting triptorelin acetate (Decapeptyl; Ferring, SaintPrex, Switzerland) on Day 3 of the cycle. After 30–42 days of downregulation, gonadotrophin (Gn, r-hFSH, Serono, Switzerland, 75 IU/day) and/or hMG (Lipo Biochem, 75 IU/day) were added for ovulation induction. When there were more than 2 follicles with a diameter of 18 mm, r-hfsh and hMG were stopped, 250 µg recombinant human chorionic gonadotrophin (r-HCG, Serono, Switzerland) was injected, and ultrasound-guided aspiration was performed 36 h late. On the 3rd to 5th day after oocyte retrieval, embryo morphology was scored according to morphological parameters [16], and high-quality embryos were selected for embryo transfer. Intrauterine transfer was performed under the guidance of ultrasound. Clinical pregnancy was defined as a positive serum hCG level with ultrasound evidence of a gestational sac at approximately 6 weeks of gestation [5].

Serum FSH, LH, E_2_, and P levels on the 3rd day of menstruation and the trigger day were measured. All samples were assessed using an FSH kit and E_2_ kit (chemiluminescence method). In the range of [1, 160] mIU/mL for FSH, the correlation coefficient (R) of the dose‒response curve was not lower than 0.9900 when fitted by the double logarithmic mathematical model. Repeatability (CV%) was not higher than 15.0%, and the difference between batches (CV%) was not higher than 20.0%. In the range of [0, 3000] pg/ml for E_2_, the absolute value of the dose‒response curve correlation coefficient (r) was ≥ 0.9900. The CV of the high- and low-concentration samples was not greater than 10% after 10 repeated tests. The CV among the three lot kits was not greater than 15% for the same sample.

Serum FSH recovery refers to the percentage of FSH recovered on the Gn initiation day compared to the FSH level on the pituitary suppression day. In this study, we used the GnFSH/bFSH ratio to represent FSH recovery, which was calculated as follows: GnFSH/bFSH = FSH on the Gn initiation day/baseline FSH level (FSH level on the pituitary suppression day).

### Outcome measures

The primary outcome was the ovarian sensitivity index (OSI) [5], which was calculated as follows:

$$\mathrm{OSI}^{5}=\text{Retrieved oocytes}\times{1000}/\text{total Gn dose}$$

### Statistical analysis

Statistical analyses were performed using EmpowerStats [17] (http://www.empowerstats.com; X&Y Solutions, Inc., Boston, MA, USA) and SPSS (version 27.0.1, Chicago, IL, USA) and GraphPad Prism (version 9.0, California, USA). Normally distributed continuous variables are expressed as the mean ± standard deviation (x¯±s), and categorical variables are expressed as percentages (%).

Continuous variables were expressed as mean ± standard deviation (SD). Comparisons among the three groups were performed using one-way analysis of variance (One-way ANOVA). If the ANOVA result showed an overall significance (*p* < 0.05), post-hoc pairwise comparisons were further conducted using the Tukey’s honestly significant difference (Tukey HSD) test (when variances were homogeneous) or the Games-Howell test (when variances were heterogeneous). Categorical variables are presented as numbers and percentages [n (%)]. The overall differences between groups were assessed using the chi-square test or Fisher’s exact test, as appropriate. If the overall test result was significant (*p* < 0.05), post hoc pairwise comparisons were conducted using chi-square tests with Bonferroni correction applied to adjust the significance level.

Multiple linear regression analysis was used to study the relationship between the OSI and GnFSH/bFSH. The relationship between the OSI and GnFSH/bFSH ratio was fitted by smooth curve fitting and the threshold effect value. A *P* value < 0.05 was considered statistically significant.

## Results

### Basic characteristics of the study population

**Fig. 1:**
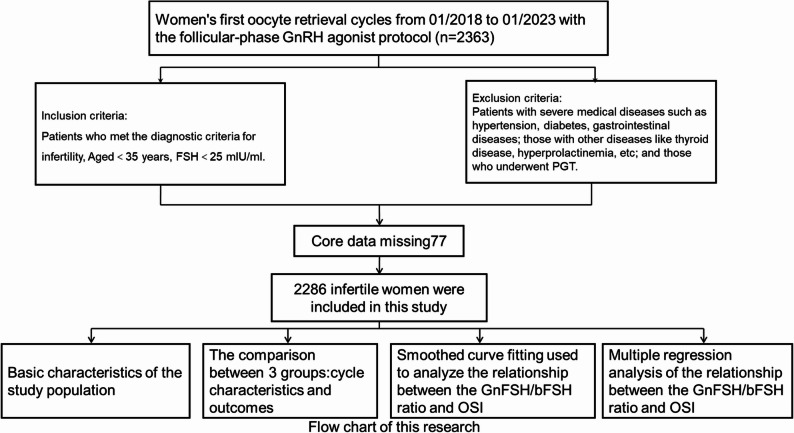
Flow chart of this research

A total of 2286 patients were included in the study. Figure 1 shows the study procedure flowchart. The median age was 29 years, the median infertility duration was 3 years, the median AMH level was 6.22 ng/ml, and the median AFC was 18. The clinical pregnancy rate in this study was 68.83%, and other basic information is shown in Table 1.

**Table 1:** Basic characteristics of the study population

Project	Value
No. of cycles	2286
Age (years)	29 (20–34)
Infertility duration (years)	3.00 (1.00–16.00)
BMI(kg/M^2^)	22.27 (15.23–35.88)
AFC	18 (5–61)
AMH(ng/ml)	6.22 (1.32–24.20)
Baseline FSH(mIU/ml)	6.59 (0.64–11.85)
Dosage of Gn (IU)	1962.50 (825.00-7762.50)
Duration of Gn (days)	12.00 (6.00–20.00)
FSH on trigger day (mIU/ml)	12.69 (4.20-37.39)
LH on trigger day (mIU/ml)	0.82 (0.00-4.97)
E_2_ on trigger day (pg/ml)	2255.00 (412.00-9275.00)
P on trigger day (ng/ml)	0.67 (0.08–2.58)
No. of oocytes retrieved	10 (1–22)
No. of mature oocytes	10 (0–20)
No. of transferred embryos	
1	767 (40.52%)
2	1126 (59.48%)
The type of embryo transferred	
Cleavage-stage embryo	534 (28.21%)
Blastocyst	1359 (71.79%)
Clinical pregnancy	
Unpregnant	590 (31.17%)
Pregnant	1303 (68.83%)

### Comparison of baseline characteristics and outcomes among the 3 groups

**Fig. 2:**
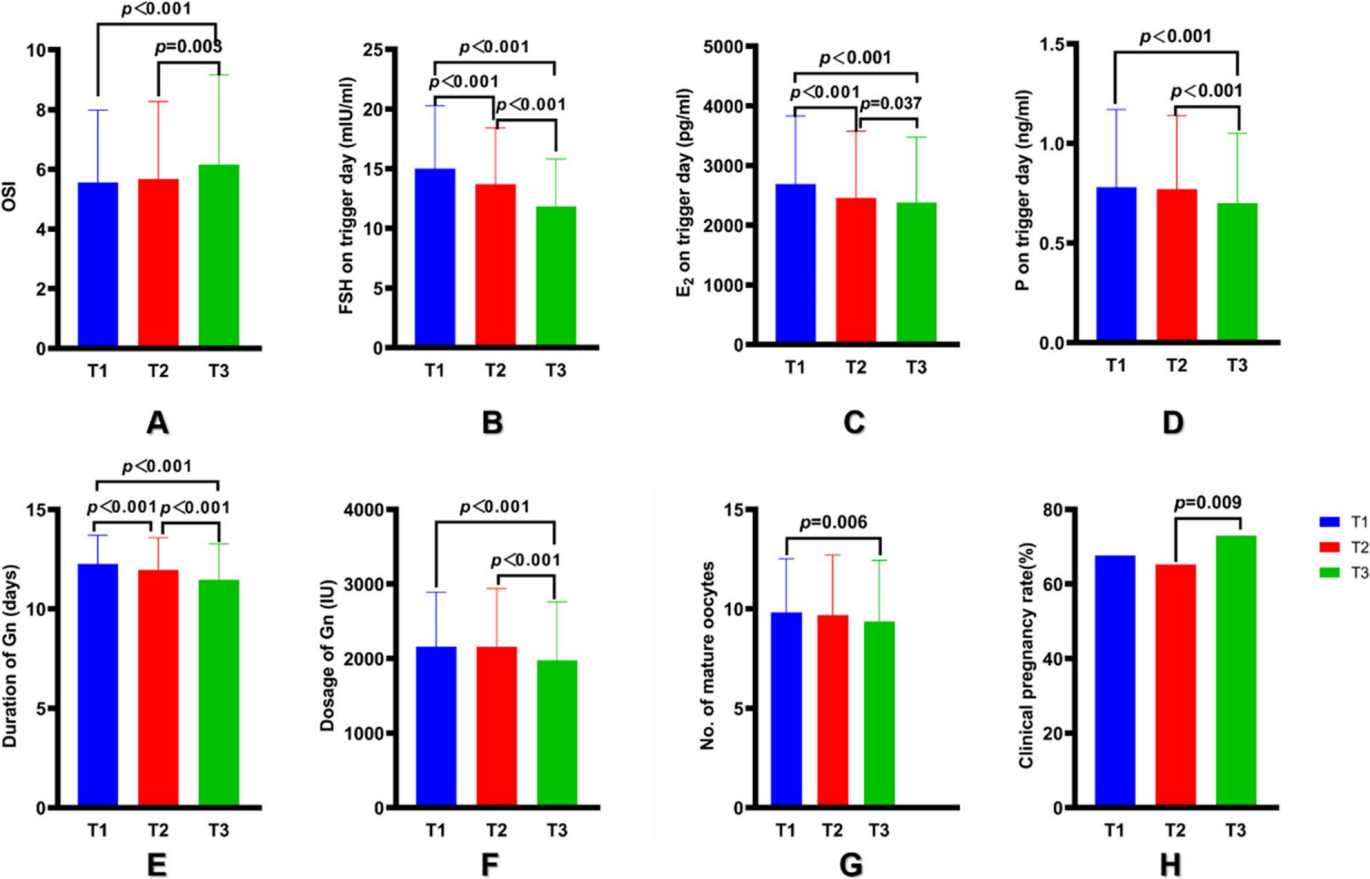
The post hoc analysis of clinical data in GnFSH/bFSH tertile groups (T1, T2, T3). **A** OSI. **B** FSH on trigger day. **C** E_2_ on trigger day. **D** P on trigger day. **E** Duration of Gn. **F** Dosage of Gn. **G** No. of mature oocytes. **H** Clinical pregnancy rate. Statistical analysis: Data for continuous variables (**A**-**G**) are presented as mean ± SD and were analyzed by one-way ANOVA followed by Tukey’s post hoc test for multiple comparisons. Data for the categorical variable (**H**) are presented as % and were compared between groups using Chi-square test, with Bonferroni correction applied for pairwise post hoc analyses. Note: OSI, ovarian sensitivity index; FSH, follicle stimulating hormone; E_2_, estradiol; P, progesterone; Gn, gonadotropin

As shown in Table 2, Patients in group T2 and T3 demonstrated higher AFC and AMH levels, and lower baseline FSH compared to the T1 group. Additionally, a slight but significant increase in maternal age was observed across the tertiles (*p* = 0.015), while the duration of infertility showed no significant difference among groups (*p* = 0.06). The distribution of infertility types (primary vs. secondary) and causes (particularly ovulatory disorders and male factor) also differed significantly among the tertiles (*p* < 0.05). BMI values showed a progressive increase from T1 to T3 groups (*p* < 0.001). OSI was significantly higher in the T3 group compared to T1 and T2 groups (Fig. 2A). Hormonal levels on the trigger day showed distinct patterns: FSH and E₂ levels decreased significantly with increasing tertiles, while progesterone (P) was significantly lower in the T3 group(*p* < 0.05) (Fig. 2B-D). The T3 group required a significantly lower total gonadotropin dosage and shorter stimulation duration(*p* < 0.05) (Fig. 2E-F). The number of mature oocytes was significantly higher in the T1 group(T1 > T3, *p* = 0.006), the clinical pregnancy rate was significantly higher in the T3 group (T2 < T3, *p* = 0.009) (Fig. 2G-H). No significant differences were observed in LH levels on trigger day, the number of oocytes retrieved, the number of embryos transferred, or the type of embryos transferred among the three groups(*p* > 0.05). Other infertility causes including pelvic/tubal factors, endometriosis, immunological and unexplained infertility showed no significant differences across the groups(*p* > 0.05). The results of post hoc pairwise comparisons are presented in Supplementary Table S1.

**Table 2:** Comparison of baseline characteristics and outcomes among the 3 groups

GROUP	T1 (<0.37)	T2 (0.37–0.56)	T3 (>0.56)	*p*-value	Effect Size (η²)	Post-hoc Analysis (Tukey HSD)
No. of cycles	762	762	762			
Continuous Variables, Mean ± SD						
Age (years)	28.69 ± 2.89	28.83 ± 2.85	29.11 ± 2.79	0.015	0.004	T1 < T3^*^
Infertility duration (years)	3.15 ± 1.99	3.33 ± 2.07	3.40 ± 2.18	0.06	0.002	
BMI(kg/M^2^)	21.29 ± 2.94	22.90 ± 3.10	23.93 ± 3.07	< 0.001	0.113	T1 < T2^*^, T1 < T3^*^, T2 < T3^*^
AFC	18.07 ± 6.68	19.79 ± 7.65	20.98 ± 7.90	< 0.001	0.024	T1 < T2^*^,T1 < T3^*^,T2 < T3^*^
AMH(ng/ml)	6.48 ± 3.43	7.27 ± 3.84	7.64 ± 4.15	< 0.001	0.015	T1 < T2^*^, T1 < T3^*^
Baseline FSH(mIU/ml)	7.31 ± 1.44	6.79 ± 1.28	5.98 ± 1.21	< 0.001		T1 > T2^*^, T1 > T3^*^, T2 > T3^*^
OSI	5.55 ± 2.43	5.68 ± 2.59	6.16 ± 3.01	< 0.001		T1 < T3^*^, T2 < T3^*^
Dosage of Gn (IU)	2158.72 ± 733.46	2155.65 ± 783.71	1975.19 ± 786.33	< 0.001	0.012	T1 > T3^*^, T2 > T3^*^
Duration of Gn (days)	12.27 ± 1.43	11.96 ± 1.62	11.47 ± 1.81	< 0.001	0.038	T1 > T2^*^, T1 > T3^*^, T2 > T3^*^
FSH on trigger day (mIU/ml)	15.01 ± 5.28	13.69 ± 4.72	11.84 ± 3.96	< 0.001	0.071	T1 > T2^*^, T1 > T3^*^, T2 > T3^*^
LH on trigger day (mIU/ml)	0.95 ± 0.55	0.93 ± 0.52	0.93 ± 0.47	0.526	0	
E_2_ on trigger day (pg/ml)	2689.76 ± 1139.20	2457.79 ± 1118.07	2380.34 ± 1094.13	< 0.001	0.013	T1 > T2^*^, T1 > T3^*^
P on trigger day (ng/ml)	0.78 ± 0.39	0.77 ± 0.37	0.70 ± 0.35	< 0.001	0.008	T1 > T3^*^, T2 > T3^*^
No. of oocytes retrieved	10.70 ± 2.77	10.78 ± 2.89	10.47 ± 2.75	0.077	0.002	
No. of mature oocytes	9.83 ± 2.69	9.69 ± 3.01	9.37 ± 3.07	0.008	0.003	T1 > T3^*^

Categorical Variables, n (%)	T1	T2	T3	*p*-value	Effect Size (φc/V)	
Infertility type, n (%)				0.036	0.054	
Primary	488 (64.21)	456 (59.84)	441 (57.87)			T1 > T3^*^
Secondary	272 (35.79)	306 (40.16)	321 (42.13)			
Infertility cause, n (%)				< 0.001	0.102	
Pelvic and tubal factors	390 (51.18)	395 (51.84)	395 (51.84)	0.957		
Ovulation disorder	121 (15.88)	171 (22.44)	204 (26.77)	< 0.001		T1 < T2^*^, T1 < T3^*^
Endometriosis	19 (2.49)	15 (1.97)	19 (2.49)	0.734		
Male factor	215 (28.22)	172 (22.57)	132 (17.32)	< 0.001		T1 > T2^*^, T1 > T3^*^, T2 > T3^*^
Immunological infertility	0 (0.00)	2 (0.26)	1 (0.13)	0.367		
Unexplained infertility	17 (2.23)	7 (0.92)	11 (1.44)	0.11		
No. of transferred embryos				0.199	0.042	
1	254 (43.05)	235 (38.59)	245 (38.70)			
2	336 (56.95)	374 (61.41)	388 (61.30)			
The type of embryo transferred				0.779	0.017	
Cleavage-stage embryo	168 (28.47)	176 (28.90)	172 (27.17)			
Blastocyst	422 (71.53)	433 (71.10)	461 (72.83)			
Clinical pregnancy				0.01	0.071	
Unpregnant	191 (32.37)	212 (34.81)	171 (27.01)			
Pregnant	399 (67.63)	397 (65.19)	462 (72.99)			T2 < T3^*^

Statistical analysis: Data for continuous variables (A-G) are presented as mean ± SD and were analyzed by one-way ANOVA followed by Tukey's post hoc test for multiple comparisons. Data for the categorical variable (H) are presented as % and were compared between groups using Chi-square test, with Bonferroni correction applied for pairwise post hoc analyses

*OSI* Ovarian sensitivity index, *FSH* Follicle stimulating hormone, *E2* Estradiol, *P* Progesterone, *Gn* Gonadotropin

^*^p<0.05

### Threshold effect of the relationship between the GnFSH/bFSH ratio and OSI

**Fig. 3:**
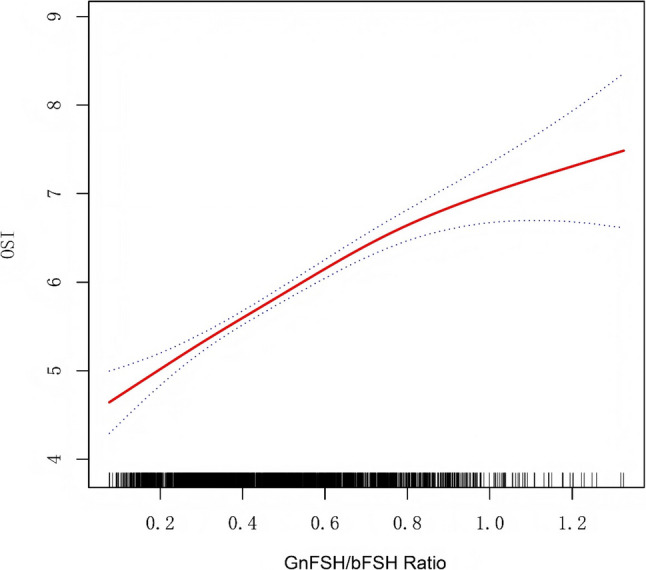
Association between GnFSH/bFSH and OSI. All adjusted for Age, BMI, AMH, AFC, infertility cause, Infertility type. Red line represents the smooth curve fit between variables and the blue dotted curves represents the 95% of confidence interval

After adjustment for potential confounders (age, BMI, AMH level, AFC, infertility cause, infertility type), the relationship between the basal GnFSH/bFSH ratio and OSI was analyzed (Fig. 3). Table 3 presents the threshold effect analysis for the association between the GnFSH/bFSH ratio and OSI. The table contains two models (Models I and II) and their respective adjusted beta coefficients (95% CI) and P values. In Model I (linear analysis), the one-line slope has an adjusted β of 2.60 with a 95% CI of (2.10, 3.09) and *P* < 0.0001. In Model II (nonlinear analysis), the turning point is 0.8, with a slope l of 2.96 (95% CI: 2.37, 3.56) and *P* < 0.0001 for values below 0.8 and a slope 2 of 0.19 (95% CI: -2.02, 2.39) and P value of 0.8686 for values above 0.8. The LRT test results indicated that there was a significant difference between Models I and II, with P values of 0.028, suggesting a nonlinear relationship between the GnFSH/bFSH ratio and OSI.

**Table 3:** Threshold effect of the relationship between the GnFSH/bFSH ratio and OSI

Models	OSI	
	Adjustedβ(95%CI)	*p* value
Models I		
One line slope	2.60 (2.10, 3.09)	< 0.0001
Models II		
Turning point	0.8	
<0.8 slope1	2.96 (2.37, 3.56)	< 0.0001
>0.8 Slope2	0.19 (-2.02, 2.39)	0.8686
LRT test		0.028

Models I, linear analysis; Models II, non-linear analysis. LRT test, Logarithmic likelihood ratio test (p value<0.05 means Models II is significantly different from Models I, which indicates a non-linear relationship). Adjusted for Age, BMI, AMH, AFC, infertility cause, Infertility type

### Multiple linear regression aof the relationship between the GnFSH/bFSH and OSI

In the multiple linear regression model, after full adjustment (for age, BMI, AMH level, AFC, infertility cause, infertility type), each one-unit increase in the GnFSH/bFSH ratio was related to increased OSI values (β = 2.59, 95% CI 2.10, 3.09, *P* < 0.0001). In GnFSH/bFSH tertile groups, patients in T2 (0.37–0.56) or T3 (> 0.56) had increased OSI values (β = 0.49, 95% CI 0.24, 0.74, *P* = 0.0001 and β = 1.25, 95% CI 0.99, 1.51, *P* < 0.0001, respectively) when compared with patients who had ratio values in T1 (< 0.37) (Table 4).

**Table 4:** Association between the GnFSH/bFSH and OSI in unadjusted and adjusted regression models

Variables	Un Adjusted Model		Adjusted Model I		Adjusted Model II	
GnFSH/bFSH	β (95%CI)	*P* value	β (95%CI)	*P* value	β (95%CI)	*P* value
Continuous	1.27 (0.76, 1.78)	< 0.0001	2.59 (2.10, 3.08)	< 0.0001	2.59 (2.10, 3.09)	< 0.0001
Categories						
T1	0		0		0	
T2	0.13 (-0.14, 0.40)	0.3437	0.48 (0.23, 0.73)	0.0002	0.49 (0.24, 0.74)	0.0001
T3	0.60 (0.33, 0.87)	< 0.0001	1.23 (0.97, 1.49)	< 0.0001	1.25 (0.99, 1.51)	< 0.0001

When the GnFSH/bFSH ratio was treated as a continuous independent variable: The Unadjusted Model was a simple linear regression without any covariates. Adjusted Model I was a multivariable linear regression adjusted for Age, BMI, AMH, and AFC. Adjusted Model II was a multivariable linear regression further adjusted for infertility cause and infertility type in addition to all covariates in Model I. The complete results of these multivariable models, including coefficients for all covariates, are presented in Supplementary Table S2–Table S6

When the GnFSH/bFSH ratio was analyzed as a categorical variable: The categorical analysis employed a multivariable linear regression framework in which the GnFSH/bFSH ratio groups were incorporated using dummy variables, with T1 serving as the reference category. The coefficients for T2 and T3 represent the adjusted mean differences in OSI relative to T1, controlling for the same sets of covariates as in the corresponding continuous model. Full results are provided in the Supplementary Material

## Discussion

Controlled ovarian hyperstimulation (COH), as an important aspect of human-assisted reproductive technology, greatly affects the outcomes of IVF/ICSI-ET. The gonadotropin-releasing hormone agonist (GnRHa) has long been used in COH due to its role in preventing premature follicular luteinization. As one of the mainstream protocols in current ovarian hyperstimulation, the follicular-phase long GnRH agonist (GnRHa) protocol has many advantages, such as good inhibition of the endogenous LH peak, synchronization of follicles and follicles, synchronization of follicle development and hormone growth, synchronization of follicle and embryo development, and synchronization of embryo development and the endometrium to improve the pregnancy rate. It can also improve endometrial receptivity by promoting apoptosis of endometrial cells and regulating immune and signaling pathways [17–19].

The follicular-phase long GnRHa protocol is the mainstream protocol in our center, which mainly uses GnRHa long-acting preparations. As with other ovarian stimulation protocols, the dosage of Gn in the follicular-phase long GnRHa protocol also needs indicators related to ovarian response. The ovarian reserve is the main factor affecting the ovarian response to COH. In the past, clinical indicators, including age, AMH level, AFC, basal FSH level and FSH/LH levels, were commonly used to reflect ovarian reserve [20, 21]. A poor ovarian response has been reported in patients with normal FSH levels [22]. Researchers have also used the combined application of AMH levels and AFC to assess ovarian response [23]. Mutlu [24] showed that AMH levels have a low sensitivity for predicting poor ovarian response. Overall, there are no specific markers to evaluate ovarian reserve and response individually, and they still need to be evaluated in combination. The results of the prospective study [6] showed that AMH levels were highly correlated with the OSI and could be used as a surrogate for AMH monitoring to predict ovarian responsiveness in patients undergoing IVF. Although the OSI has been recognized by many scholars to reflect ovarian response, it cannot provide clinicians with reference data before the initiation of ovarian stimulation. Therefore, the purpose of our study was to discover indicators that can predict ovarian response before the initiation of ovarian stimulation and that are closely related to the OSI.

In the follicular-phase long GnRHa protocol, after full-dose depot GnRH-an injection, the level of sex hormones could be completely suppressed for 2 weeks, and FSH levels gradually recovered after 3–4 weeks [25]. In our study, all subjects received a GnRHa injection on the third day of menstruation, and the FSH level on the day of GnRHa injection was called the basal FSH level. Gn was given 30–42 days after GnRHa injection, and the FSH level on the day of Gn was called GnFSH. Since FSH levels gradually recovered after 3–4 weeks of downregulation, the GnFSH/bFSH ratio indicates the recovery of endogenous FSH, and the correlation between the recovery of endogenous FSH and the OSI has not been reported until now.

In our study, we found that the GnFSH/bFSH ratio was positively correlated with the OSI. Yamashita [26], who studied the effect of the FSH/LH ratio on IVF-ET outcomes after pituitary downregulation, found that the FSH/LH ratio was positively correlated with the Gn dosage after pituitary downregulation. He also noted that the recovery of LH spontaneous release and stimulated release was slow and dose dependent, while the recovery of the FSH concentration and stimulated release was faster. As he evaluated the FSH/LH ratio, this study cannot confirm his view; however, his study suggested that the recovery of FSH after pituitary downregulation may affect the Gn dosage. De Ziegler D [27] also mentioned that FSH and LH release after pituitary downregulation by GnRHa suggested restoration of endogenous GnRH action in the pituitary gland. Hastie [28] conducted animal experiments with sheep and found that insulin-like growth factor binding proteins (IGFBPs) are involved in the regulation of IGF-I and IGF-II, thereby regulating the growth and development of ovine follicles. GnRHa administration may affect follicle development by changing the proliferation and differentiation of IGFBP levels in ovine follicles through changes in FSH and LH levels. These results suggest that the changes in FSH levels after pituitary downregulation may be related to follicular development and ovarian response.

Considering that there are many factors affecting the OSI, after adjusting for other confounding factors, we used a smooth fitting curve and threshold saturation effect analysis and found that when the GnFSH/bFSH ratio was less than 0.8, the OSI level gradually increased with the recovery of endogenous FSH. In the process of COH with the follicular-phase prolonged protocol, the use of GnRH-a to downregulate pituitary function can reduce the circulating endogenous follicle stimulating hormone (FSH) level below the threshold to support follicular growth [29], and then supraphysiological doses of Gn can be administered to obtain more synchronous follicular development. However, both endogenous and exogenous FSH contribute to follicle growth during this process. The GnFSH/bFSH ratio indicates the percentage of endogenous FSH to basal FSH during pituitary recovery after downregulation. We can imagine that the total FSH level during COH ovulation is the sum of endogenous FSH and exogenous FSH. Assuming that the total FSH level is relatively constant, the higher the endogenous FSH level is, the lower the exogenous FSH level will be. The level of exogenous FSH is closely related to the dosage of Gn, so it determines the OSI. The ovarian sensitivity index (OSI) has been suggested as a measurement of ovarian sensitivity during the IVF procedure, which could reflect the potential of follicles to produce oocytes in response to exogenous Gn stimulation [30, 31]. This may also explain why the OSI was increased with a GnFSH/bFSH ratio < 0.8.

## Conclusion

We found that after pituitary suppression, the recovery of serum FSH (GnFSH/bFSH) is an independent risk factor for increased OSI values. When the GnFSH/bFSH ratio was less than 0.8, the OSI increased with the increase in the GnFSH/bFSH ratio value. However, when the ratio was higher than 0.8, there was no significant relationship between the GnFSH/bFSH ratio and OSI. This conclusion provides some basis for clinicians to adjust the dosage of Gn during COH. However, our study has certain limitations. For example, we derived our findings from a relatively small number of individuals, which should be validated in a larger cohort of Chinese patients. Second, this study was limited by its retrospective nature and single center design. In the future, the sample size will be increased, or a multicenter study will be performed for further validation.

## Supplementary Information

Supplementary Material 1.

## Data Availability

All data presented in this study are available upon request upon contact with the corresponding author.

## Abbreviations

COH: Controlled ovarian hyperstimulation
GnRHa: Gonadotropin-releasing hormone analog agonist
hCG: Human chorionic gonadotrophin
IVF: In vitro fertilization
ET: Embryo transfer
FSH: Follicle-stimulating hormone
Gn: Gonadotropin
