# Supplementary material for: Does the recovery of serum FSH influence the ovarian sensitivity index in the follicular-phase depot GnRH agonist protocol?
Source: BMC Pregnancy Childbirth. 2026 May 19;26:751. doi: 10.1186/s12884-026-09246-3 (PMC13353009; doi:10.1186/s12884-026-09246-3)
Supplement: Supplementary file 1 — Supplementary Material 1. [file 12884_2026_9246_MOESM1_ESM.docx]

**Supplementary**

**Table S1** Comparisons of Continuous and Categorical Variables Across Groups, with Post Hoc Analyses Using Tukey HSD and Bonferroni Correction.

| **GROUP** | **T1** | **T2** | **T3** | ***p-value*** | **Effect Size (η²)** | **Post-hoc Analysis (Tukey HSD)** | **p-values between two groups** | | |
| --- | --- | --- | --- | --- | --- | --- | --- | --- | --- |
|  | **(＜0.37)** | **(0.37-0.56)** | **(＞0.56)** |  |  |  | **T1 vs T2** | **T1 vs T3** | **T2 vs T3** |
| No. of cycles | 762 | 762 | 762 |  |  |  |  |  |  |
| Continuous Variables, Mean ± SD |  |  |  |  |  |  |  |  |  |
| Age (years) | 28.69 ± 2.89 | 28.83 ± 2.85 | 29.11 ± 2.79 | 0.015 | 0.004 | T1<T3* | 0.611 | 0.012 | 0.138 |
| Infertility duration (years) | 3.15 ± 1.99 | 3.33 ± 2.07 | 3.40 ± 2.18 | 0.06 | 0.002 |  | 0.221 | 0.06 | 0.793 |
| BMI（kg/M^2^） | 21.29 ± 2.94 | 22.90 ± 3.10 | 23.93 ± 3.07 | <0.001 | 0.113 | T1<T2*,T1<T3*,T2<T3* | <0.001 | <0.001 | <0.001 |
| AFC | 18.07 ± 6.68 | 19.79 ± 7.65 | 20.98 ± 7.90 | <0.001 | 0.024 | T1<T2*,T1<T3*,T2<T3* | <0.001 | <0.001 | 0.008 |
| AMH（ng/ml） | 6.48 ± 3.43 | 7.27 ± 3.84 | 7.64 ± 4.15 | <0.001 | 0.015 | T1<T2*,T1<T3* | <0.001 | <0.001 | 0.18 |
| Baseline FSH(mIU/ml) | 7.31 ± 1.44 | 6.79 ± 1.28 | 5.98 ± 1.21 | <0.001 |  | T1>T2*,T1>T3*,T2>T3* | <0.001 | <0.001 | <0.001 |
| OSI | 5.55 ± 2.43 | 5.68 ± 2.59 | 6.16 ± 3.01 | <0.001 |  | T1<T3*,T2<T3* | 0.568 | <0.001 | 0.003 |
| Dosage of Gn (IU) | 2158.72 ± 733.46 | 2155.65 ± 783.71 | 1975.19 ± 786.33 | <0.001 | 0.012 | T1>T3*,T2>T3* | 0.997 | <0.001 | <0.001 |
| Duration of Gn (days) | 12.27 ± 1.43 | 11.96 ± 1.62 | 11.47 ± 1.81 | <0.001 | 0.038 | T1>T2*,T1>T3*,T2>T3* | <0.001 | <0.001 | <0.001 |
| FSH on trigger day (mIU/ml) | 15.01 ± 5.28 | 13.69 ± 4.72 | 11.84 ± 3.96 | <0.001 | 0.071 | T1>T2*,T1>T3*,T2>T3* | <0.001 | <0.001 | <0.001 |
| LH on trigger day (mIU/ml) | 0.95 ± 0.55 | 0.93 ± 0.52 | 0.93 ± 0.47 | 0.526 | 0 |  |  |  |  |
| E_2_ on trigger day (pg/ml) | 2689.76 ± 1139.20 | 2457.79 ± 1118.07 | 2380.34 ± 1094.13 | <0.001 | 0.013 | T1>T2*,T1>T3* | <0.001 | <0.001 | 0.37 |
| P on trigger day (ng/ml) | 0.78 ± 0.39 | 0.77 ± 0.37 | 0.70 ± 0.35 | <0.001 | 0.008 | T1>T3*,T2>T3* | 0.765 | <0.001 | <0.001 |
| No. of oocytes retrieved | 10.70 ± 2.77 | 10.78 ± 2.89 | 10.47 ± 2.75 | 0.077 | 0.002 |  |  |  |  |
| No. of mature oocytes | 9.83 ± 2.69 | 9.69 ± 3.01 | 9.37 ± 3.07 | 0.008 | 0.003 | T1>T3* | 0.643 | 0.006 | 0.094 |
| Categorical Variables, n (%) | **T1** | **T2** | **T3** | ***p*-value** | **Effect Size (φc/V)** |  | **corrected p-values between two groups (raw p-values)** | | |
| Infertility type, n (%) |  |  |  | 0.036 | 0.054 |  | 0.246(0.082) | 0.036(0.012) | 1.000(0.435) |
| Primary | 488 (64.21) | 456 (59.84) | 441 (57.87) |  |  | T1>T3* |  |  |  |
| Secondary | 272 (35.79) | 306 (40.16) | 321 (42.13) |  |  |  |  |  |  |
| Infertility cause, n (%) |  |  |  | <0.001 | 0.102 |  |  |  |  |
| Pelvic and tubal factors | 390 (51.18) | 395 (51.84) | 395 (51.84) | 0.957 |  |  |  |  |  |
| Ovulation disorder | 121 (15.88) | 171 (22.44) | 204 (26.77) | <0.001 |  | T1<T2*,T1<T3* | 0.003(0.001) | <0.003(<0.001) | 0.150(0.05) |
| Endometriosis | 19 (2.49) | 15 (1.97) | 19 (2.49) | 0.734 |  |  |  |  |  |
| Male factor | 215 (28.22) | 172 (22.57) | 132 (17.32) | <0.001 |  | T1>T2*,T1>T3*,T2>T3* | 0.033(0.011) | <0.003(<0.001) | 0.030(0.010) |
| Immunological infertility | 0 (0.00) | 2 (0.26) | 1 (0.13) | 0.367 |  |  |  |  |  |
| Unexplained infertility | 17 (2.23) | 7 (0.92) | 11 (1.44) | 0.11 |  |  |  |  |  |
| No. of transferred embryos |  |  |  | 0.199 | 0.042 |  |  |  |  |
| 1 | 254 (43.05) | 235 (38.59) | 245 (38.70) |  |  |  |  |  |  |
| 2 | 336 (56.95) | 374 (61.41) | 388 (61.30) |  |  |  |  |  |  |
| The type of embryo transferred |  |  |  | 0.779 | 0.017 |  |  |  |  |
| Cleavage-stage embryo | 168 (28.47) | 176 (28.90) | 172 (27.17) |  |  |  |  |  |  |
| Blastocyst | 422 (71.53) | 433 (71.10) | 461 (72.83) |  |  |  |  |  |  |
| Clinical pregnancy |  |  |  | 0.01 | 0.071 |  | 1.000(0.372) | 0.120(0.040) | 0.009(0.003) |
| Unpregnant | 191 (32.37) | 212 (34.81) | 171 (27.01) |  |  |  |  |  |  |
| Pregnant | 399 (67.63) | 397 (65.19) | 462 (72.99) |  |  | T2<T3* |  |  |  |

Notes: Continuous variables are presented as mean ± SD and compared using one-way ANOVA with Tukey HSD post hoc test. Categorical variables are presented as n (%) and compared using the chi-square or Fisher's exact test, with Bonferroni correction for pairwise comparisons.

**Table S2 Results of univariable linear regression analyses examining the associations between various factors and OSI**

| Variable | Unstandardized Coefficient (β) | Standard Error | t-value | p-value | 95% CI |
| --- | --- | --- | --- | --- | --- |
| Intercept | 5.183 | 0.139 | 37.376 | < 0.001 | 4.911,5.455 |
| GnFSH/bFSH ratio | 1.27 | 0.262 | 4.853 | < 0.001 | 0.757,1.783 |
| Intercept | 5.197 | 0.149 | 34.919 | < 0.001 | 4.905,5.489 |
| group3(T1,T2,T3) | 0.301 | 0.069 | 4.362 | < 0.001 | 0.165,0.436 |
| Intercept | 9.263 | 0.572 | 16.205 | < 0.001 | 8.142,10.384 |
| Age | -0.12 | 0.02 | -6.091 | < 0.001 | -0.159,-0.081 |
| Intercept | 10.573 | 0.389 | 27.17 | < 0.001 | 9.81,11.337 |
| BMI | -0.21 | 0.017 | -12.385 | < 0.001 | -0.243,-0.177 |
| Intercept | 4.575 | 0.116 | 39.342 | < 0.001 | 4.347,4.803 |
| AMH | 0.173 | 0.014 | 12.024 | < 0.001 | 0.144,0.201 |
| Intercept | 4.512 | 0.155 | 29.047 | < 0.001 | 4.207,4.817 |
| AFC | 0.066 | 0.007 | 8.881 | < 0.001 | 0.051,0.08 |
| Intercept | 5.835 | 0.171 | 34.173 | < 0.001 | 5.5,6.17 |
| Infertility Type | -0.026 | 0.116 | -0.229 | 0.819 | -0.253,0.2 |
| Intercept | 5.679 | 0.104 | 54.46 | < 0.001 | 5.474,5.883 |
| Infertility Cause | 0.059 | 0.043 | 1.366 | 0.172 | -0.026,0.144 |

Notes: Each variable was analyzed in a separate univariable linear regression model.

**Table S3** Full Results of the Adjusted Model I for GnFSH/bFSH ratio and OSI (Multivariable Linear Regression)

| Variable | Unstandardized Coefficient (β) | Standard Error | t-value | p-value | 95% CI |
| --- | --- | --- | --- | --- | --- |
| Intercept | 14.038 | 0.633 | 22.162 | < 0.001 | 12.796,15.28 |
| GnFSH/bFSH ratio | 2.59 | 0.25 | 10.35 | < 0.001 | 2.099,3.08 |
| Age | -0.129 | 0.018 | -7.215 | < 0.001 | -0.164,-0.094 |
| BMI | -0.344 | 0.018 | -19.654 | < 0.001 | -0.379,-0.31 |
| AMH | 0.129 | 0.017 | 7.53 | < 0.001 | 0.095,0.162 |
| AFC | 0.058 | 0.009 | 6.361 | < 0.001 | 0.04,0.076 |

**Table S4** Full Results of the Adjusted Model II for GnFSH/bFSH ratio and OSI (Multivariable Linear Regression)

| Variable | Unstandardized Coefficient(β) | Standard Error | t-value | p-value | 95% CI |
| --- | --- | --- | --- | --- | --- |
| Intercept | 13.734 | 0.651 | 21.102 | < 0.001 | 12.457,15.01 |
| GnFSH/bFSH ratio | 2.59 | 0.25 | 10.343 | < 0.001 | 2.099,3.081 |
| Age | -0.131 | 0.018 | -7.267 | < 0.001 | -0.167,-0.096 |
| BMI | -0.345 | 0.018 | -19.686 | < 0.001 | -0.379,-0.31 |
| AMH | 0.128 | 0.017 | 7.488 | < 0.001 | 0.094,0.161 |
| AFC | 0.059 | 0.009 | 6.453 | < 0.001 | 0.041,0.077 |
| Infertility Type | 0.174 | 0.106 | 1.648 | 0.1 | -0.033,0.381 |
| Infertility Cause | 0.061 | 0.039 | 1.554 | 0.12 | -0.016,0.138 |

**Table S5** Full Results of the Adjusted Model I for GnFSH/bFSH tertile groups and OSI (Multivariable Linear Regression)

| Variable | Unstandardized Coefficient(β) | Standard Error | t-value | p-value | 95% CI |
| --- | --- | --- | --- | --- | --- |
| Intercept | 13.777 | 0.635 | 21.712 | < 0.001 | 12.533,15.022 |
| group3(T1,T2,T3) | 0.618 | 0.066 | 9.355 | < 0.001 | 0.488,0.747 |
| T1 | 0 |  |  |  |  |
| T2 | 0.481155 | 0.127478 | 3.774 | 0.000165 | 0.23, 0.73 |
| T3 | 1.232714 | 0.132105 | 9.331 | < 0.001 | 0.97, 1.49 |
| Age | -0.125 | 0.018 | -6.971 | < 0.001 | -0.16,-0.09 |
| BMI | -0.337 | 0.018 | -19.227 | < 0.001 | -0.371,-0.302 |
| AMH | 0.129 | 0.017 | 7.538 | < 0.001 | 0.096,0.163 |
| AFC | 0.058 | 0.009 | 6.284 | < 0.001 | 0.04,0.076 |

**Table S6** Full Results of the Adjusted Model II for GnFSH/bFSH tertile groups and OSI (Multivariable Linear Regression)

| Variable | Unstandardized Coefficient(β) | Standard Error | t-value | p-value | 95% CI |
| --- | --- | --- | --- | --- | --- |
| Intercept | 13.447 | 0.652 | 20.627 | < 0.001 | 12.169,14.726 |
| group3(T1,T2,T3) | 0.621 | 0.066 | 9.385 | < 0.001 | 0.491,0.751 |
| T1 | 0 |  |  |  |  |
| T2 | 0.48984 | 0.12747 | 3.843 | 0.000125 | 0.24, 0.74 |
| T3 | 1.25215 | 0.13228 | 9.466 | < 0.001 | 0.99, 1.51 |
| Age | -0.127 | 0.018 | -7.026 | < 0.001 | -0.163,-0.092 |
| BMI | -0.338 | 0.018 | -19.273 | < 0.001 | -0.372,-0.303 |
| AMH | 0.129 | 0.017 | 7.491 | < 0.001 | 0.095,0.162 |
| AFC | 0.059 | 0.009 | 6.384 | < 0.001 | 0.041,0.077 |
| Infertility Type | 0.182 | 0.106 | 1.714 | 0.087 | -0.026,0.39 |
| Infertility Cause | 0.068 | 0.039 | 1.72 | 0.086 | -0.01,0.145 |
